# Association between daily use of social media and behavioral lifestyles in the Saudi community: a cross-sectional study

**DOI:** 10.3389/fpubh.2023.1254603

**Published:** 2023-10-09

**Authors:** Nasser F. BinDhim, Nora A. Althumiri, Rashed Abdullah Al-Duraihem, Saeed Alasmary, Zaied Alkhamaali, Abdulhameed Abdullah Alhabeeb

**Affiliations:** ^1^Informed Decision-Making for Research and Studies, Riyadh, Saudi Arabia; ^2^National Center for Mental Health Promotion, Riyadh, Saudi Arabia; ^3^Saudi Food and Drug Authority, Riyadh, Saudi Arabia

**Keywords:** social media, Saudi Arabia, behavior, lifestyle, mental health

## Abstract

**Objective:**

This study aimed to investigate the association between nine social media platforms use and health-related behavior, including fruit and vegetable intake, physical activity, tobacco use, and risk factors including depression and obesity.

**Methods:**

A cross-sectional study was conducted using secondary data from the Sharik Health Indicators Surveillance System (SHISS). Participants 18 years and older were recruited via phone-interviews. The nine social media platform use [Twitter-(X), Facebook, Instagram, WhatsApp, LinkedIn, Snapchat, TikTok, Telegram, and YouTube] were assessed using self-reported use. Health-related variables include behavioral factors including diet, physical activity, and tobacco use including (cigarettes, waterpipes, and e-cigarettes), risk of depression and obesity. Logistic regression analysis was performed to explore the association between social media use and health-related variables.

**Results:**

The study indicated that daily Snapchat users had a lower healthy diet (fruit and vegetable intake), whereas daily LinkedIn and WhatsApp users were positively associated with a healthier diet, relative to those with infrequent social media use. Furthermore, daily interaction with Instagram, TikTok, Telegram, and YouTube was significantly associated with increased depression risk. Conversely, Snapchat and WhatsApp usage was significantly linked to a decreased depression risk. Tobacco-smoking behaviors were associated with specific social media platforms: cigarette smoking was associated with Snapchat, TikTok, and YouTube; e-cigarette with Facebook, LinkedIn, Snapchat, and TikTok; and waterpipe smoking with Facebook and TikTok. Interestingly, some platforms, such as Instagram, were associated with reduced cigarette smoking. The relationship between social media activity and health-related outcomes remained significant after adjusting for age and gender.

**Conclusion:**

This study highlights the potential negative impact of particular daily social media use on health-related variables, including dietary habits, tobacco use, and depression. Nevertheless, particular daily social media use of some platforms was associated with a potential positive impact on the health-related variables. Social media platforms are tools that can be used to achieve both a positive and negative effect. By knowing which demographic segments have a greater presence on one platform, we are creating opportunities to understand the social phenomena and at the same time use it to reach those segments and communicate with them, because each social media platform has its unique way and framework of user communication.

## 1. Background

Over the last 20 years, social media has emerged from obscurity to become a fully integrated part of everyday life (1). The United States has one of the highest social network usage rates in the world (1). In 2020, over 223 million Americans were using social networks to send private messages, post pictures, or like or post comments on content posted by others (2). Facebook is the largest social media platform in the world, with over 2.9 billion users (3). Other social media platforms, including YouTube and WhatsApp, also have more than 2 billion users each (4). These numbers are huge, considering that there are 7.7 billion people in the world, at least 3.5 billion of whom have access to the internet. This means that social media platforms are now used by a third of the people in the world and by more than two-thirds of all internet users (4). In the United States, more females use social media than males (54 and 46%, respectively); males account for 54.4% of the average global population (5). Meanwhile, the number of social media users in Saudi Arabia is also on the rise (6). According to a report from the BBC, among Middle Eastern countries, Saudi Arabia has the largest social media user base (6). Due to the high rate of smartphone use in the country, 79.3% of the Saudi population are active social media users, and 27.66 million of them access it via mobile device (6). These users are most active on social media platforms such as Twitter, YouTube, and Facebook (7). In this regard, it is mainly religious, political, and cultural factors that shape the social media engagement of the country (8). These factors influence Saudi nationals to use social media platforms in different contexts (8). This offers them ways to interact and communicate, and also helps in promoting user engagement (9).

Research into the effects of social media on health and behavior in general has expanded rapidly in recent years (10). In addition, it been encouraged by controversial public debates and critical primary research, systematic reviews, and meta-analyses (10). These works focus on the usage of social media and its links to health and behavior, especially for young people (11). For instance, a cohort study of 6,595 teenagers aged 12–15 years in the United States found that teenagers who spend more than 3 h per day using social media might be at a higher risk for mental health problems compared with those who never use it (12).

In contrast, other research suggests that social media may even have health related and behavioral lifestyle benefits (13). Recent discourse around the implications of social media on health and behavioral lifestyles has predominantly spotlighted its potential drawbacks. Such apprehensions often concern issues related to mental health, privacy, and cyberbullying (14). However, an emerging body of research provides a counter-narrative, positing that social media, when used judiciously, can offer tangible benefits for both mental and physical well-being.

Specifically, studies have shown that social media platforms can serve as tools for disseminating health-related information, facilitating peer support, and even aiding in chronic disease management (15). For instance, online communities provide venues for patients to share their experiences, which often leads to better coping mechanisms and improved mental health outcomes (16). Furthermore, these platforms have also been employed in public health campaigns, with agencies leveraging their wide reach to spread awareness about diseases, vaccinations, and health-promoting behaviors (17).

Another facet where social media has demonstrated potential benefits is in the realm of behavioral lifestyles. Platforms such as Instagram and YouTube are full with fitness influencers and health educators who disseminate advice on exercise routines, balanced diets, and mindfulness practices (18). For many, these platforms serve as primary sources of inspiration and motivation for adopting healthier lifestyles (11).

Nonetheless, it is imperative to approach these findings with caution. The quality of information on social media platforms is heterogeneous, and users need to be discerning about their sources. Furthermore, while certain studies underscore the benefits of social media, it is crucial to balance this perspective with research highlighting its potential drawbacks, ensuring a comprehensive understanding of its overall impact on health and behavioral lifestyle (19).

These conflicting findings make it challenging to navigate the research investigating the effects of social media on mental health and how best to use social media. There is evidence that, in general, routine social media use is positively associated with health and social well-being. However, this is only the case if the user is not “emotionally invested” in the media; if they are, the outcomes are negative (12). Nevertheless, the degree to which social media harms health or behavior is debatable. Another recent study found that most studies investigating the link between social media and mental health demonstrate “weak” or “inconsistent” associations (12).

Thus, the objective of the this research project was to explore the association between the utilization of nine distinct social media platforms, namely Twitter (X), Facebook, Instagram, WhatsApp, LinkedIn, Snapchat, TikTok, Telegram, and YouTube, and health-related behaviors. These behaviors encompassed consumption patterns of fruits and vegetables, engagement in physical activities, usage of tobacco, and potential risk factors such as depression risk and obesity.

## 2. Methods

### 2.1. Design

This study comprises a secondary analysis of cross-sectional data from the Sharik Health Indicators Surveillance System (SHISS) (20). The SHISS consists of short phone interviews conducted from all 13 administrative regions of Saudi Arabia on a quarterly basis. Each interview lasts approximately 8–12 min and is conducted by a trained data collector. In SHISS, the ZdataCloud® system was used. It has integrated eligibility and sampling modules, which control the distribution of the sample and prevent human-related sampling bias (21). All questions had to be answered for an individual questionnaire to be successfully submitted to the database. All data were coded, collected and stored through the ZdataCloud® system and database (21). The Sharik institutional review board granted approval for this project (approval no. 2021–2), in line with Saudi Arabia’s research ethics standards and regulations.

### 2.2. Sampling and sample size

The SHISS achieves an equal distribution of participants, stratified by age and gender, within and across the 13 regions of Saudi Arabia, by using a proportional quota sampling. It employs two age groups, based on the Saudi Arabian median age of 36 years, which generates a quota of 52 for this study. Researchers calculated the sample size based on a medium effect size of nearly 0.3, with 80% power and a 95% confidence level, allowing for comparison between quotas based on region, age, and gender (16). Consequently, each quota necessitates at least 135 participants and total sample was 7,020 participants. The ZdataCloud® stops accepting participants with similar characteristics once the quota sample is reached. The data collection system, with no human interference, automatically controls the quota sampling process (15).

### 2.3. Participants and recruitment

The study focused on recruiting Arabic-speaking Saudi residents aged 18 or older. The Sharik Association for Research and Studies (formerly known as the Sharik Association for Health Research) provided a random list of mobile phone numbers to identify possible participants (17). The Sharik database contains individuals interested in taking part in future research projects, with over 170,000 users spread across Saudi Arabia’s 13 regions and still expanding (17). Participants were called up to three times, and if there was no response, another individual with similar demographics was selected from the database. This process continued until the required number of participants was reached and the recruitment process was automatically closed. Consent was verbally acquired from participants during the interview sessions. Once consent was given, the interviewer evaluated the participant’s eligibility, and if they met the criteria, the interview commenced.

### 2.4. Questionnaire design and validation

The SHISS dataset includes behavioral risk factors (diet, physical activity, and tobacco use, including cigarettes, waterpipes, and e-cigarettes), the risk of depression, which is measured using the Patient Health Questionnaire (PHQ-2) with a cut-off of 3 and above (22, 23), and obesity, measured as the body mass index (BMI) via height and weight. We calculated the participants’ body mass indices (BMIs). We used the Center for Disease Control and Prevention’s (CDC) BMI category status, which classifies a BMI of 30 or above as obese (24). By the end of 2021, the SHISS included a variable for collecting data related to the daily use of some social media applications, including Twitter (X), Facebook, Instagram, WhatsApp, LinkedIn, Snapchat, TikTok, Telegram, and YouTube.

This study also used the WHO’s global recommendations of physical activity for adults (18–64 years old): (1) vigorous-intensity physical activity (VIPA) for 75 min per week, or (2) moderate-intensity physical activity (MIPA) for 150 min per week (25). Based on the participants’ self-reported responses to the interview questionnaire (i.e., the weekly number of minutes, frequency, and intensity level of exercise), two categorical outcome variables were created that reflected whether or not the guidelines were met: an acceptable level of physical activity (ALPA; at least 150 min of MIPA per week and/or at least 75 min of VIPA per week) and a low level of physical activity (LLPA; less than 150 min of MIPA and/or less than 75 min of VIPA).

We asked the participants about their daily fruit and vegetable intake. If a participant’s daily food intake included at least one portion of fruit and one portion of vegetables, they were categorized as having an acceptable level of fruit and vegetable intake (AFVI). If not, they were categorized as having a low level of fruit and vegetable intake (LFVI).

Sharik Health Indicators Surveillance System questions were linguistically validated to ensure that the participant understood the questions as intended and could provide accurate answers. The validation was preformed via focus group, asked to discuss and respond to the survey as one group. This process was repeated with the same people to test the survey’s reliability. Afterwards, a new group was interviewed to ensure the clarity of the original meaning and to develop modified questions. The final version of the questionnaire was produced accordingly.

### 2.5. Statistical analysis

Data were transferred to the Statistical Package for Social Sciences (SPSS), version 25, which was used for data management and analyses. This study employed automated electronic data collection, there were no missing values; the ZdataCloud involves a data integrity check to prevent users from entering invalid data (e.g., the minimum age is 18) (21). Descriptive statistics were used to describe the variables. The quantitative variables are presented herein as the mean and SD if they have a normal distribution, or as the median and range, as appropriate. The categorical variables are presented as percentages. Logistic crude regression, adjusted for age and gender, was used to was used to explore the association between social media use and other health-related variables.

## 3. Results

In total, between July and December of 2021, 13,915 participants from the 13 administrative regions of Saudi Arabia completed the interview, with a response rate of 67.29%. Out of the total contacted potential participants, 49.9% were female. The mean age was 36.80 (SD: 13.68; minimum: 18; maximum: 99; range: 81). Table 1 shows the demographic distribution of participants according to their daily social media usage.

**Table 1:** Demographic distribution of participants (*n* = 13,915) in the context of their daily social media usage.

	Twitter (X)	Facebook	Instagram	WhatsApp	LinkedIn	Snapchat	TikTok	Telegram	YouTube
	*n* (%)	*n* (%)	*n* (%)	*n* (%)	*n* (%)	*n* (%)	*n* (%)	*n* (%)	*n* (%)
Sex									
Male	2,893 (41.5)	481 (6.9)	1839 (26.4)	4,861 (69.7)	216 (3.1)	3,895 (54.4)	1,699 (24.4)	819)11.7(	2,194 (31.5)
Female	2,283 (32.9)	265 (3.8)	2,563 (36.9)	4,695 (67.6)	124 (1.8)	4,410 (63.5)	1927 (27.8)	851)12.3(	1,611 (23.2)
Age									
≤19	288 (40.9)	23 (3.3)	378 (53.7)	422 (59.9)	9 (1.3)	501 (71.2)	302 (42.9)	122 (17.3)	269 (38.2)
20–29	2,160 (46.8)	176 (3.8)	2014 (43.6)	2,904 (62.9)	168 (3.6)	3,365 (72.9)	1,551 (33.6)	723 (15.7)	1,614 (35.0)
30–39	1,174 (41.4)	208 (7.3)	967 (34.1)	2040 (71.9)	85 (3.0)	1854 (65.4)	744 (26.2)	350 (12.3)	793 (28.0)
40–49	1,000 (33.2)	183 (6.1)	700 (23.3)	2,240 (74.5)	53 (1.8)	1,597 (53.1)	620 (20.6)	325 (10.8)	637 (21.2)
50–59	445 (24.3)	122 (6.7)	278 (15.2)	1,393 (76.0)	18 (1.0)	705 (38.4)	306 (16.7)	125 (6.8)	351 (19.1)
≥60	109 (11.9)	34 (3.7)	65 (1.5)	557 (60.9)	7 (0.8)	183 (20.0)	103 (11.3)	23 (2.5)	141 (15.4)
Level of education									
Lower than a bachelor’s degree	2,079 (28.6)	348 (4.8)	2,064 (28.4)	4,851 (66.8)	74 (1.0)	4,023 (55.4)	1,995 (27.5)	687 (9.5)	1,926 (26.5)
Bachelor’s degree and above	3,097 (46.5)	398 (6.0)	2,338 (35.1)	4,705 (70.7)	266 (4.0)	4,182 (62.8)	1,631 (24.5)	983 (14.8)	1,879 (28.2)
Region									
Asir	390 (36.0)	39 (3.6)	368 (34.0)	770 (71.2)	14 (1.3)	687 (63.5)	320 (29.6)	156 (10.2)	319 (29.5)
Baha	475 (43.9)	56 (5.2)	348 (32.1)	725 (66.9)	24 (2.2)	584 (53.9)	320 (29.6)	111 (10.2)	290 (32.3)
Eastern region	364 (33.7)	55 (5.1)	467 (43.2)	816 (75.5)	44 (4.1)	647 (59.9)	188 (17.4)	139 (12.9)	349 (32.3)
Hail	418 (38.6)	52 (5.1)	276 (25.5)	692 (64.0)	32 (3.0)	628 (58.8)	326 (30.1)	141 (13.0)	266 (24.6)
Jazan	262 (24.2)	65 (6.0)	273 (25.2)	750 (69.)	8 (0.7)	599 (55.3)	347 (32.0)	147 (13.6)	347 (32.0)
Al Jouf	406 (37.6)	38 (3.5)	284 (26.3)	772 (71.5)	10 (0.9)	676 (62.6)	282 (26.1)	97 (9.0)	214 (19.8)
Madinah	450 (41.4)	59 (5.4)	417 (38.3)	755 (69.4)	30 (2.8)	669 (61.5)	259 (23.8)	138 (12.7)	338 (31.1)
Makkah	328 (30.3)	106 (9.8)	378 (35.0)	779 (72.1)	48 (4.4)	628 (58.1)	258 (23.9)	121 (11.2)	350 (32.4)
Najran	250 (27.1)	27 (2.9)	169 (18.3)	506 (54.9)	4 (0.4)	390 (42.3)	117 (12.7)	36 (3.9)	124 (13.4)
Northern border	458 (42.4)	44 (4.1)	336 (31.1)	718 (66.4)	27 (2.5)	658 (60.9)	346 (32.0)	136 (12.6)	301 (27.8)
Qassim	468 (43.3)	76 (7.0)	324 (29.9)	762 (70.4)	34 (3.1)	706 (65.2)	275 (25.4)	142 (13.1)	282 (26.1)
Riyadh	464 (42.8)	74 (6.8)	392 (36.1)	767 (70.7)	48 (4.4)	661 (60.9)	279 (25.7)	141 (13.0)	313 (28.8)
Tabuk	443 (40.8)	55 (5.4)	370 (34.1)	744 (68.6)	17 (1.6)	672 (61.9)	292 (26.9)	165 (15.2)	312 (28.8)
Overall	5,176 (37.2)	746 (5.4)	4,402 (31.6)	9,556 (68.7)	340 (2.4)	8,205 (59.0)	3,626 (6.1)	1,670 (12.0)	3,805 (27.3)

Table 2 shows a crosstabulation between daily social media platform use and health-related behaviors including obesity, physical activity, fruit and vegetable intake, smoking (traditional cigarettes, waterpipes, and e-cigarette), and the risk of depression.

**Table 2:** Cross tabulation between daily use of social media platforms and health-related behaviors.

	Twitter (X)	Facebook	Instagram	WhatsApp	LinkedIn	Snapchat	TikTok	Telegram	YouTube
	*n* (%)	*n* (%)	*n* (%)	*n* (%)	*n* (%)	*n* (%)	*n* (%)	*n* (%)	*n* (%)
Obesity									
Yes	921 (17.8)	201 (26.9)	820 (18.6)	2,273 (23.8)	61 (17.9)	1713 (20.9)	719 (19.8)	307 (18.4)	780 (20.5)
No	4,255 (25.2)	545 (22.2)	3,582 (19.6)	7,283 (67.5)	279 (22.6)	6,492 (24.8)	2,907 (23.4)	1,363 (23.0)	3,025 (23.2)
Physical activity									
MPA	1,082 (20.9)	146 (19.6)	876 (19.9)	1958 (20.5)	71 (20.9)	1,652 (20.1)	694 (19.1)	351 (21.0)	772 (20.3)
VPA	783 (15.1)	119 (16.0)	628 (14.3)	1,271 (13.3)	57 (16.8)	1,156 (14.1)	509 (14.0)	238 (14.3)	578 (15.2)
Combined	1,367 (26.4)	194 (26.0)	1,108 (25.2)	2,414 (25.3)	96 (28.2)	2075 (25.3)	889 (24.5)	440 (26.3)	989 (26.0)
Fruit and vegetable Intake									
ALF	582 (11.2)	106 (14.2)	488 (11.1)	1,261 (13.2)	54 (15.9)	900 (11.0)	405 (11.2)	210 (12.6)	437 (11.5)
ALV	612 (11.8)	115 (15.4)	512 (11.6)	1,330 (13.9)	57 (16.8)	990 (12.1)	450 (12.4)	220 (11.6)	462 (12.1)
Combined	290 (5.6)	57 (7.6)	226 (5.1)	632 (6.6)	31 (9.1)	416 (5.1)	193 (5.3)	99 (5.9)	220 (5.8)
Smoking									
Cigarettes	1,059 (20.5)	191 (25.6)	718 (16.3)	1,751 (18.3)	96 (28.2)	1,518 (18.5)	720 (19.9)	295 (11.7)	819 (21.5)
E-cigarettes	765 (14.8)	135 (18.1)	626 (14.2)	1,103 (11.5)	87 (25.6)	1,132 (13.8)	534 (14.7)	237 (14.2)	628 (16.5)
Waterpipes	907 (17.5)	188 (25.2)	716 (16.3)	1,451 (15.2)	88 (25.9)	1,358 (16.6)	627 (17.3)	264 (15.8)	710 (18.7)
Risk of depression (PHQ-2)									
At risk (cut-off 3 and above)	1,114 (21.5)	160 (21.4)	1,050 (23.9)	1,893 (19.8)	90 (26.5)	1,727 (21.0)	889 (24.5)	424 (25.4)	881 (23.2)
Not at risk	4,062 (78.5)	586 (78.6)	3,352 (76.1)	7,663 (80.3)	250 (73.5)	6,478 (79.0)	2,737 (24.7)	1,246 (74.6)	2,924 (76.8)

Table 3 shows an association between the daily use of social media platforms and health-related behaviors including obesity, physical activity, fruit and vegetable intake, smoking (traditional cigarettes, waterpipes, and e-cigarettes), and the risk of depression, compared to those who do not use these platforms on a daily basis.

**Table 3:** Logistic regression results of the association between daily use of social media platforms and health-related variables, compared to those who do not use these platforms on a daily basis.

Variable	Crude OR (95% CI) (*p* value)	Adjusted OR (95% CI) (*p* value)
Obesity		
Twitter (X)	0.68 (0.62–0.75) (<0.001)	0.80 (0.73–0.88) (<0.001)
Facebook	1.53 (0.1.29–1.82) (<0.001)	1.4 (1.16–1.66) (<0.001)
Instagram	0.79 (0.72–0.88) (<0.001)	0.900 (0.81–1.00) (0.050)
WhatsApp	1.400 (1.27–1.53) (<0.001)	1.26 (1.15–1.38) (<0.001)
LinkedIn	0.88 (0.66–1.17) (0.38)	0.96 (0.72–1.29) (<0.001)
Snapchat	0.91 (0.83–0.98) (0.027)	1.11 (1.01–1.22) (0.03)
TikTok	0.89 (0.83–0.98) (0.036)	0.96 (0.87–1.06) (<0.001)
Telegram	0.88 (0.76–1.01) (0.08)	0.90 (0.78–1.04) (0.16)
YouTube	0.98 (0.90–1.1) (0.74)	1.08 (0.97–1.19) (0.14)
Physical activity (combined)		
Twitter (X)	1.13 (1.04–1.23) (0.003)	1.07 (0.98–1.17) (0.095)
Facebook	1.02 (0.86–1.22) (0.747)	0.98 (0.83–1.17) (0.902)
Instagram	0.96 (0.88–1.06) (0.487)	1.01 (0.92–1.12) (0.696)
WhatsApp	1.08 (0.99–1.17) (0.075)	1.08 (0.99–1.17) (0.080)
LinkedIn	1.12 (0.87–1.43) (0.355)	1.06 (0.83–1.36) (0.600)
Snapchat	1.08 (0.97–1.15) (0.178)	1.07 (0.98–1.17) (0.090)
TikTok	0.93 (0.84–1.02) (0.147)	0.94 (0.85–1.03) (0.200)
Telegram	1.074 (0.96–1.21) (0.242)	1.03 (0.90–1.17) (0.620)
YouTube	1.08 (0.99–1.18) (0.069)	0.99 (0.90–1.09) (0.880)
Healthy diet (combined)		
Twitter (X)	0.89 (0.76–1.04) (0.174)	1.02(0.86–1.20) (0.805)
Facebook	1.26 (0.94–1.69) (0.109)	1.26 (0.84–1.50) (0.426)
Instagram	0.83 (0.70–0.99) (0.047)	0.98 (0.81–1.17) (0.835)
WhatsApp	1.31 (1.12–1.53) (<0.001)	1.20 (1.03–1.41) (0.020)
LinkedIn	1.26 (1.12–2.47) (0.010)	1.77 (1.19–2.63) (0.004)
Snapchat	0.63 (0.55–0.74) (<0.001)	0.77 (0.66–0.91) (0.002)
TikTok	0.91 (0.76–1.09) (0.333)	0.97 (0.81–1.17) (0.817)
Telegram	1.03 (0.81–1.30) (0.786)	1.06 (0.84–1.35) (0.583)
YouTube	0.99 (0.83–1.18) (0.979)	1.04 (0.87–1.24) (0.666)
Smoking cigarettes		
Twitter (X)	1.12 (0.99–1.25) (0.052)	0.898 (0.79–1.01) (0.083)
Facebook	1.40 (1.13–1.73) (0.002)	1.09 (0.87–1.37) (0.420)
Instagram	0.59 (0.52–0.68) (<0.001)	0.84 (0.73–0.98) (0.029)
WhatsApp	1.01 (0.90–1.14) (0.750)	0.92 (0.81–1.05) (0.234)
LinkedIn	1.31 (0.95–1.79) (0.092)	1.09 (0.79–1.51) (0.580)
Snapchat	1.03 (0.92–1.16) (0.582)	1.31 (1.15–1.49) (<0.001)
TikTok	1.15 (1.02–1.3) (0.023)	1.27 (1.11–1.45) (<0.001)
Telegram	0.88 (0.73–1.05) (0.162)	0.95 (0.79–1.15) (0.663)
YouTube	1.47 (1.30–1.67) (<0.001)	1.14 (1.00–1.30) (0.044)
Smoking e-cigarettes		
Twitter (X)	1.29 (1.10–1.51) (0.002)	1.09 (0.93–1.28) (0.266)
Facebook	1.66 (1.28–2.15) (<0.001)	1.50 (1.14–1.96) (0.003)
Instagram	0.86 (0.72–1.03) (0.100)	1.03 (0.86–1.24) (0.680)
WhatsApp	0.76 (0.65–0.89) (0.001)	0.75 (0.64–0.89) (0.001)
LinkedIn	2.00 (1.42–2.82) (<0.001)	1.77 (1.25–2.51) (0.001)
Snapchat	1.20 (1.02–1.42) (0.026)	1.26 (1.06–1.49) (0.009)
TikTok	1.16 (1.42–2.82) (0.070)	1.24 (1.04–1.47) (0.014)
Telegram	0.91 (0.72–1.14) (0.422)	0.94 (0.74–1.19) (0.62)
YouTube	1.47 (1.24–1.74) (<0.001)	1.17 (0.99–1.40) (0.065)
Smoking waterpipes		
Twitter (X)	1.19 (1.01–1.40) (0.032)	1.01 (0.86–1.20) (0.830)
Facebook	2.11 (1.63–2.73) (<0.001)	1.75 (1.34–2.28) (<0.001)
Instagram	0.74 (0.61–0.89) (0.002)	0.99 (0.81–1.20) (0.949)
WhatsApp	0.83 (0.70–0.98) (0.027)	0.76 (0.64–0.90) (0.001)
LinkedIn	1.46 (0.97–2.18) (0.063)	1.29 (0.86–1.93) (0.217)
Snapchat	0.911 (0.77–1.074) (0.267)	1.08 (0.91–1.08) (0.342)
TikTok	1.21 (1.01–1.44) (0.034)	1.31 (1.10–1.57) (0.003)
Telegram	0.91 (0.71–1.16) (0.472)	0.97 (0.75–1.25) (0.834)
YouTube	1.35 (1.12–1.61) (0.001)	1.11 (0.92–1.33) (0.255)
At risk of depression (PHQ-2)		
Twitter (X)	0.98 (0.89–1.07) (0.687)	0.99 (0.90–1.08) (0.839)
Facebook	0.89 (0.74–1.07) (0.231)	0.98 (0.81–1.18) (0.842)
Instagram	1.24 (1.13–1.37) (<0.001)	1.12 (1.01–1.24) (0.025)
WhatsApp	0.81 (0.74–0.89) (<0.001)	0.85 (0.77–0.93) (<0.001)
LinkedIn	1.20 (0.93–1.55) (0.149)	1.24 (0.96–1.60) (0.092)
Snapchat	0.96 (0.87–1.05) (0.391)	0.88 (0.80–0.96) (0.009)
TikTok	1.30 (1.18–1.43) (<0.001)	1.25 (1.14–1.38) (<0.001)
Telegram	1.24 (1.08–1.41) (0.001)	1.21 (1.06–1.38) (0.004)
YouTube	1.11 (1.10–1.23) (0.034)	1.16 (1.04–1.28) (0.004)

In terms of obesity, seven out of nine social media platforms show a significant association with obesity after adjusting for age and gender, including Twitter, Facebook, WhatsApp, LinkedIn, Snapchat, and TikTok. However, none of the social media platforms were associated with physical activity. Meanwhile, three platforms were associated with a healthy diet: LinkedIn, WhatsApp (positive association), and Snapchat (negative association).

Regarding smoking behavior, three social media platforms were positively associated with cigarette smoking: Snapchat, TikTok, and YouTube. Four social media platforms were positively associated with e-cigarette smoking, namely, Facebook, LinkedIn, Snapchat, and TikTok. Meanwhile, two social media platforms were positively associated with waterpipe smoking (Facebook and TikTok).

Finally, three social media platforms, TikTok, Telegram, and YouTube, were positively associated with a risk of depression.

## 4. Discussion

The use of social media has become increasingly prevalent in recent years, with billions of people around the world using various platforms to connect with others, share information, and engage in a variety of activities. While social media has many potential benefits, including increased social connectedness and access to information, there are also concerns about the potential negative effects of social media use on health and behaviors. In this study, we aimed to explore the association between the daily use of social media and health and behavior in the Saudi population during in the second half of 2021.

Our results showed that there was a significant association between social media use and obesity, with seven out of nine social media platforms showing a significant association with obesity. This finding is consistent with previous research that has found a link between social media use and obesity (26). One possible explanation for this association is that social media use may lead to a sedentary lifestyle and unhealthy eating habits, which can contribute to weight gain and obesity (27).

Interestingly, we did not find any significant association between social media use and physical activity. This finding is somewhat surprising, as previous research has suggested that social media use may have a negative impact on physical activity levels (28). However, it is possible that the lack of association in our study is due to the fact that we only looked at daily social media use and did not take into account other factors that may influence physical activity levels, such as work or family obligations.

We also found that three social media platforms were associated with a healthy diet, namely, LinkedIn, WhatsApp (positively), and Snapchat (negatively). This finding is consistent with previous research that has found a link between social media use and dietary habits (29). One possible explanation for this association is that social media use may influence food choices and eating behaviors through exposure to food-related content and social norms (30).

In terms of tobacco-use behaviors, we found that three social media platforms were positively associated with cigarette smoking: Snapchat, TikTok, and YouTube. E-cigarette smoking was positively associated with four social media platforms, including Facebook, LinkedIn, Snapchat, and TikTok. Finally, two social media platforms were positively associated with waterpipe smoking (Facebook and TikTok). These findings are consistent with previous research that has found a link between social media use and tobacco use (31, 32). One possible explanation for this association is that social media use may expose individuals to pro-tobacco content and social norms that promote smoking (33).

Finally, we found that three social media platforms were positively associated with the risk of depression: TikTok, Telegram, and YouTube. This finding is consistent with previous research that has found a link between social media use and depression (34). One possible explanation for this association is that social media use may lead to social comparisons and feelings of inadequacy, which can contribute to the development of depression (35).

Overall, our study provides important insights into the association between social media use and health and behaviors in the Saudi population. While our findings are consistent with previous research, it is important to note that the relationship between social media use and health and behavior is complex and multifaceted. There are many factors that can influence the impact of social media use on health and behavior, including individual differences, social norms, and cultural factors (36).

However, via studying the social media distribution among demographic variables and health indicators, and by going deeper to explore the relationship between social media daily use and specific health indicators, we can confidently lean toward the logical argument that social media platforms are tools that can be used to achieve both a positive and negative effect.

Furthermore, the way the users use these tools influences the type of individuals they attract and the way those individuals are affected by them. This can be inferred by the way, social media platforms accumulate specific demographic segments with similar health indicators. These platforms are more likely not the cause of the existence or accumulation of these segments of the population, but the way the users use these tools can be associated with the segments they attract. By knowing which segments have a greater presence on one platform, we are creating opportunities to understand the social phenomena and at the same time use it to reach those segments and communicate with them, because each social media platform has its unique way and framework of user communication.

One limitation of our study is that it is cross-sectional in nature, which means that we cannot establish causality between social media use and health and behaviors. Future research should use longitudinal designs to better understand the temporal relationship between social media use and health and behavior. Additionally, future research should explore the mechanisms underlying the association between social media use and health and behavior, including the role of social norms, social comparison, and exposure to pro-health or unhealthy content.

In conclusion, our study provides important insights into the association between social media use and health and behavior in the Saudi population. Our findings suggest that social media use is associated with a range of health and behavior outcomes, including obesity, dietary habits, tobacco use, and depression. These findings have important implications for public health interventions aimed at reducing the negative impact of social media use on health and behavior. By understanding the factors that influence the impact of social media on users’ health and behavior, we can develop more effective interventions to promote healthy behaviors and prevent negative health outcomes.

## Data availability statement

The original contributions presented in the study are included in the article/supplementary material, further inquiries can be directed to the corresponding author.

## Ethics statement

The studies involving humans were approved by Sharik institutional review board. The studies were conducted in accordance with the local legislation and institutional requirements. Written informed consent for participation was not required from the participants or the participants’ legal guardians/next of kin because the study used phone interviews. Participant consent was obtained verbally during the interviews and recorded in the data collection system.

## Author contributions

NB: Conceptualization, Data curation, Formal Analysis, Funding acquisition, Investigation, Methodology, Project administration, Software, Supervision, Validation, Writing – original draft, Writing – review & editing. NA: Conceptualization, Investigation, Methodology, Writing – original draft, Writing – review & editing. RA-D: Writing – review & editing. SA: Writing – review & editing. ZA: Writing – review & editing. AA: Writing – review & editing.
